# Determinants of institutional delivery service utilization in Ethiopia: a population based cross sectional study

**DOI:** 10.1186/s12889-020-09125-2

**Published:** 2020-07-08

**Authors:** Yebelay Berelie, Dawit Yeshiwas, Leltework Yismaw, Muluneh Alene

**Affiliations:** 1 Department of Statistics, Debre Markos University, Debre Markos, Ethiopia; 2 Department of Public Health, Debre Markos University, Debre Markos, Ethiopia

**Keywords:** Ethiopia, Institutional delivery, Multilevel, Maternal mortality, EDHS

## Abstract

**Background:**

The incidence of maternal mortality remains unacceptably high in developing countries. Ethiopia has developed many strategies to reduce maternal and child mortality by encouraging institutional delivery services. However, only one-fourth of women gave birth at health facility, in the country. This, this study aimed to identify individual level factors and to assess the regional variation of institutional delivery utilization in Ethiopia.

**Methods:**

Data were obtained from the 2016 Ethiopian demographic and health survey. In this study, a total of 7174 reproductive age women who had birth within five years were included. We fitted multilevel logistic regression model to identify significantly associated factors associated with institutional delivery. A mixture chi-square test was used to test random effects. Statistical significance was declared at *p* < 0.05, and we assessed the strength of association using odds ratios with 95% confidence intervals.

**Result:**

The level of institutional delivery was 38.9%. Women’s who had focused antenatal care (FANC) visit (AOR = 3.12, 95% CI: 2.73–3.56), multiple gestations (AOR = 2.06, 95% CI: 1.32–3.21, and being urban residence (AOR = 7.18, 95% CI: 5.10–10.12) were more likely to give birth at health facility compared to its counterpart. Compared to women’s without formal education, giving birth at health facility was more likely for women’s who had primary education level (AOR = 1.77, 95% CI: 1.49–2.10), secondary education level (AOR = 3.79, 95% CI: 2.72–5.30), and higher education level (AOR = 5.86, 95% CI: 3.25–10.58). Furthermore, women who reside in rich (AOR = 2.39, 95% CI: 1.86–3.06) and middle (AOR = 1.66, 95% CI: 1.36–2.03) household wealth index were more likely to deliver at health facility compared to women’s who reside poor household wealth index. Moreover, this study revealed that 34% of the total variation in the odds of women delivered at health institution accounted by regional level.

**Conclusion:**

The level of institutional delivery in Ethiopia remains low. Context specific and tailored programs that includes educating women and improving access to ANC services has a potential to improve institutional delivery in Ethiopia.

## Background

Maternal health refers to the health of women during pregnancy, delivery, and postpartum period. Despite maternal mortality ratio declined from 1990 to 2015, the incidence of maternal mortality remains unacceptably high in developing countries, which accounted for 99% of global maternal deaths [1]. Maternal mortality has been high in sub-Saharan Africa and South Asia. Sub-Saharan Africa alone accounts 56% of maternal mortality with Maternal Mortality Ratio (MMR) 500 per 100, 000 live births [2]. Ethiopia is the one of developing countries with a high maternal mortality rate in Sub-Saharan Africa [3]. According to the World Health Organization (WHO) 2010 report, nearly 9000 maternal deaths occurred in the country [4]. The consecutive Ethiopian Demographic and Health Survey (EDHS) reports showed that MMR was 871 per 100,000 live birth in 2000, 673 per 100,000 live births in 2005, 676 per 100,000 live births in 2011, and 412 per 100,000 live births in 2016 [2, 3, 5, 6].

Evidences showed that about 66% of all maternal deaths worldwide and over 50% in developing countries were directly related to unsafe delivery practice [7, 8]. Institutional delivery service has been encouraged to improve maternal and child health [9, 10]. Although it is known that skilled health professionals are key actors to reduce maternal mortality by preventing and managing complications during pregnancy and childbirth, still a number of women’s are died due to giving birth without the attendance of skilled health worker [3, 11, 12]. Additionally, institutional delivery utilization and postnatal services are important for the survival and well-being of mother and neonates.

According to WHO, all women needs access to health services such as prenatal visits, skilled birth attendant and postnatal care visits [11]. Even though, high proportion of women had received antenatal care services [13], only one in three women are utilized institutional delivery, in developing countries [14]. Delivery care practice differs with respect to residence, culture, availability and accessibility of the health care services [15]. Increasing institutional delivery is the central goal of the safe motherhood and child survival movements [16]. If the institutional delivery care service is available and used by all women, a reduction of maternal mortality can be achieved.

Despite a number of studies were conducted and many strategies have been developed to improve the use of maternal health services in Ethiopia, yet only one-fourth of women are delivered at health facility [5]. In addition, a descriptive analysis of the 2016 EDHS showed that variations in utilization of institutional delivery were observed by region, place of residence, wealth index and women’s education level [5]. However, there is a need to explore more on factors associated with institutional delivery in Ethiopia by incorporating the hierarchical structure of data in the population. Addressing regional disparities in accessing maternal health care services should be regarded as priority to reducing the maternal mortality in Ethiopia. Additionally, the target groups should be identified for specific interventions using some advanced statistical method. Thus, this study aimed to identify the individual and regional level determinants of institutional delivery in Ethiopia.

## Methods

### Data extraction

The data for this study were obtained from the 2016 EDHS, which is collected between January 18 and June 27, 2016. The EDHS was designed to provide health and demographic estimates in nine geographical regions and two administrative cities. A total of 15,683 women of age 15–49 were interviewed in the survey. We excluded women who did not have a live birth in the five-year period prior to the survey (*n* = 8509). Therefore, the analytic sample for the current study consisted of 7174 women who had at least one live birth in the last five years prior to the survey. The 2016 EDHS dataset has a hierarchical structure as women are nested within geographical regions. The hierarchy for this study follows individuals as level-1 and regions as level-2.

### Variables

Utilization of institutional delivery was the outcome variable of the current study. At the time of survey, women were asked whether they were delivered at health facility or not. We created a binary response variable and coded as 1 for institutional delivery, 0 otherwise. Independent variables considered in this study were selected based on existing literature. These include socio-demographic factors (age of the women at birth, place of residence, distance to health facility, women’s education levels, sex of household head, wealth index, religion, employment status), and pregnancy related factors (antenatal care visits, parity, birth type, child size, desire of pregnancy).

### Statistical analysis

Descriptive statistics were performed to describe and summarize the data. In the bivariate analysis, we conducted cross-tabulation between each independent variable and place of delivery. Chi-square test and *p*-values were used to test for the significance of each factor. Further analyses were carried out to study the relationship between the independent variables and place of delivery in a multivariate setup. The impacts of collinearity among the variables were detected using VIF and tolerance. The VIF values of all independent variables in the model are below 5, which shows there is no multicollinearity problem symptoms. To properly account the hierarchical nature of the data and to avoid possible under-estimation of parameters from a single-level model, we fitted multilevel model [17]. In the current study, we consider region of residence and wealth index as the level-2 variable under which the respondents are nested. This approach extends from single level logistic regression model by including random effects from the model. Three models namely model 1 a random intercept model where the model is fitted with only the intercept, model 2 with only individual-level factors and model 3 with the effects of individual-level and region level factors were estimated. Hence, the log of the probability of utilization of institutional delivery was modeled using two-level multilevel model as follows:

$$ \log \left(\frac{\pi_{ij}}{1-{\pi}_{ij}}\right)={\beta}_0+\sum \limits_{k=1}^{\mathrm{n}}{\beta}_k{x}_{kij}+{u}_{0j}+\sum \limits_{p=1}^{\mathrm{m}}{u}_{pj}{x}_{pij} $$

where *π*_*ij*_ is probability of utilization of institutional delivery and 1- *π*_*ij*_ is probability of home delivery. The first part of the equation, $$ {\beta}_0+{\sum}_{k=1}^{10}{\beta}_k{x}_{kij} $$, is called the fixed part of the model and the second part $$ {u}_{0j}+{\sum}_{p=1}^2{u}_{pj}{x}_{pij} $$ is called the random part. The distribution of *u*_0*j*_ is normal with mean 0 and variance $$ {\sigma}_{\mathrm{u}0}^2 $$ and also the distribution of regional effect variables *u*_*pj*_ is normal with mean 0 and $$ \mathrm{variance}{\sigma}_{up}^2 $$. The intra-class correlation coefficient (ICC) measures the proportion of variance in the outcome explained by the grouping structure. It can be calculated as: $$ \mathrm{ICC}=\frac{\sigma_{u0}^2}{\sigma_{u0}^2+{\sigma}_e^2} $$ where, $$ {\sigma}_e^2 $$ is variance of individual level units which is constant as $$ \raisebox{1ex}{${\uppi}^2$}\!\left/ \!\raisebox{-1ex}{$3$}\right.\approx 3.29 $$ in logistic regression. Statistical significance was considered at *p* < 0.05 and the strength of statistical association was assessed by adjusted odds ratios (AOR) with 95% confidence intervals.

## Results

### Descriptive results of predictor variables with institutional delivery

Of the 7174 women included in this study, 36.3% of the respondents had visited health facilities for ANC at least four times during their last pregnancy. About 66.9% of women who had at least four ANC visits were delivered at health institution. Higher proportion (78.8%) of women are resides in rural areas, and only one-fourth (26.9%) of them delivered their most recent birth at a health facility. Similarly, large proportion of women had no formal education (60.6%). Only 4.4% of the women had attended higher level of education. More than half (53.2%) of women resides in households with poor wealth index. About 93.6% of women reported that their most recent pregnancy was intended. About 43.3% of the women were unemployed. Distance from their home to health facility was a big challenge for more than 56% of the respondents, and of which, only 37% of women were discharge their recent birth in health facility. Detail descriptions of the study participants is presented in (Table 1).

**Table 1:** Descriptive statistics of the study sample by status of institutional delivery (*n* = 7174)

Variables with their category	Total n (%)	Institutional delivery		Chisq. (*P*-value)
		No n(%)	Yes n(%)	
ANC use				
Less than 4 visits	4573(63.7)	3523(77.0)	1050(23.0)	1344.54 (< 0.001)
4 or more visits	2601(36.3)	862(33.1)	1739(66.9)	
Age at birth				
15–24	1913(26.7)	1082(56.6)	831(43.4)	51.43 (< 0.001)
25–34	3470(48.3)	2089(60.2)	1381(39.8)	
35–49	1791(25)	1214(67.8)	577(32.2)	
Place of residence				
Rural	5653(78.8)	4131(73.1)	1522(26.9)	1603.10 (< 0.001)
Urban	1521(21.2)	254(16.7)	1267(83.3)	
Educational level				
Not educated	4346(60.6)	3328(76.6)	1018(23.4)	1401.70 (< 0.001)
Primary	1938(27)	926(47.8)	1012(52.2)	
Secondary	575(8)	110(19.1)	575(80.9)	
Higher	315(4.4)	21(6.7)	294(93.3)	
Sex of household head				
Female	1588(22.1)	902(56.8)	686(43.2)	16.04 (< 0.001)
Male	5586(77.9)	3483(62.4)	2103(37.6)	
Parity				
First	1379(19.2)	566(41.0)	813(59.0)	477.36 (< 0.001)
2–4	3170(44.2)	1836(57.9)	1334(42.1)	
5^+^	2625(36.6)	1983(75.5)	642(24.5)	
Birth type				
Single	7033(98)	4320(61.4)	2713(38.6)	13.66 (< 0.001)
Multiple	141(2)	65(46.1)	76(53.9)	
Child size				
Large	2122(29.6)	1191(56.1)	931(43.9)	42.29 (0.001)
Average	2924(40.8)	1781(60.9)	1141(39.1)	
Small	2128(29.7)	1413(66.4)	715(33.6)	
Wealth index				
Poor	3814(53.2)	2822(74.0)	992(26.0)	585.03 (< 0.001)
Middle	1225(17.1)	627(51.2)	598(48.8)	
Rich	2135(29.8)	936(43.8)	1199(56.2)	
Religion				
Orthodox	2361(32.9)	1084(45.9)	1277(54.1)	355.25 (< 0.001)
Catholic	49(0.7)	33(67.3)	16(32.7)	
Protestant	1336(18.6)	888(66.5)	448(33.5)	
Muslim	3316(46.2)	2287(69.0)	1029(31.0)	
Others	112(1.6)	93(83.0)	19(17.0)	
Desire of Pregnancy				
Yes	6714(93.6)	4083(60.8)	2631(39.2)	4.24 (0.04)
No	460(6.4)	302(65.7)	158(34.3)	
Employment status				
Employed	4069(56.7)	2659(65.3)	1410(34.7)	70.60 (< 0.001)
Unemployed	3105(43.3)	1726(55.6)	1379(44.4)	
Distance to health facility				
Big problem	4025(56.1)	2537(63.0)	1488(37.0)	14.04 (< 0.001)
Not Big problem	3149(43.9)	1848(58.7)	1301(41.3)	
Regions				
Tigray	764(10.6)	266(6.1)	498(17.9)	1355.66 (< 0.001)
Afar	645(9.0)	556(12.7)	89(3.2)	
Amhara	763(10.6)	539(12.3)	224(8.0)	
Oromia	1030(14.4)	772(17.6)	258(9.3)	
Somali	803(11.2)	642(14.6)	161(5.8)	
Benishangul	576(8.0)	394(9.0)	182(6.5)	
SNNPR	891(12.4)	581(13.2)	310(11.1)	
Gambela	533(7.4)	326(7.4)	207(7.4)	
Harari	410(5.7)	163(3.7)	247(8.9)	
Addis Ababa	375(5.2)	15(0.3)	360(12.9)	
Dire Dawa	384(5.4)	131(3.0)	253(9.1)	

**Fig. 1:**
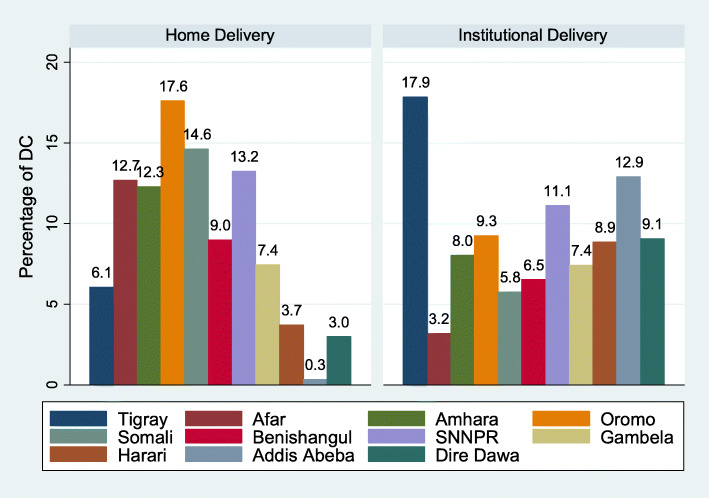
The percentage of institutional and home delivery by region

In this study, about 38.9% women delivered their most recent birth in a health institution. Among those who delivered at health institution, large proportions of women (17.9%) were in Tigray region followed by Addis Ababa city (12.9%) (Fig. 1). Women from the Afar region had the lowest institutional delivery services (3.2%) (Table 1).

### Multivariable multilevel analysis

In the multivariable analysis, the fitted models along with the estimated effects and their standard errors are presented in (Table 2). As shown in the empty model, 34% of the total variance in the odds of women delivered at health institution was accounted by grouping the women with respect to their geographical regions (ICC = 0.34). The total variance accounted by the between region in the odds of giving birth at health institution in intercept only model (model 2) and random coefficient model (model 3) were 9.2% (ICC = 0.092) and 11% (ICC = 0.11) respectively (Table 2).

**Table 2:** Multilevel Logistic Regression results of factors associated with institutional delivery among women (age 15–49) from the 2016 Ethiopian Demographic and Health survey (*n* = 7174)

Variables	Institutional delivery care		
	Model 1	Model 2	Model 3
	Odds Ratio(95%CI)	Odds Ratio(95%CI)	Odds Ratio(95%CI)
**Use of ANC**			
Less than 4 (Ref)			
4 or more		3.12(2.73,3.56)	3.12(2.73,3.56)
**Age at birth**			
15–24(Ref)			
25–34		0.91(0.76,1.08)	0.88(0.74,1.06)
35–49		0.94(0.74,1.20)	0.91(0.72,1.16)
**Place of residence**			
Rural (Ref)			
Urban		6.65(5.46,8.08)	7.18(5.10,10.12)
**Educational level**			
Not educated (Ref)			
Primary		1.76(1.51,2.04)	1.77(1.49,2.10)
Secondary		3.67(2.76,4.88)	3.79(2.72,5.30)
Higher		5.61(3.33,9.43)	5.86(3.25,10.58)
**Sex of household head**			
Female (Ref)			
Male		0.93(0.79,1.07)	0.92(0.78,1.08)
**Parity**			
First (Ref)			
2–4		0.59(0.49,0.72)	0.60(0.49,0.73)
5^+^		0.50(0.39,0.64)	0.52(0.41, 0.67)
**Birth type**			
Single (Ref)			
Multiple		2.02(1.30,3.15)	2.06(1.32,3.21)
**Child size**			
Large (Ref)			
Average		0.80(0.69,0.93)	0.80(0.68,0.92)
Small		0.70(0.60,0.83)	0.71(0.60,0.83)
**Wealth index**			
Poor (Ref)			
Middle		1.65(1.39,1.97)	1.66(1.36,2.03)
Rich		2.22(1.90,2.60)	2.39(1.86, 3.06)
**Religion**			
Orthodox (Ref)			
Catholic		0.45(0.20,0.99)	0.44(0.20,0.94)
Protestant		0.62(0.49,0.79)	0.64(0.50,0.0.82)
Muslim		1.07(0.88,1.33)	1.14(0.92,1.41)
Others		0.64(0.35,1.18)	0.62(0.34,1.15)
**Desire of pregnancy**			
Yes (Ref)			
No		1.07(0.83,1.38)	1.07(0.83,1.38)
**Employment status**			
Employed (Ref)			
Unemployed		1.05(0.92,1.20)	1.03(0.90,1.18)
Distance to healthfa			
Big Problem (Ref)			
Not big problem		1.14(1.01, 1.29)	1.13(1.06, 1.22)
**Intercept**	0.81(0.38,1.76)	0.34(0.22,0.54)	0.31(0.19,0.50)
Estimation of Random effect			
Between-region variance,	1.68(0.72,3.97)	0.33(0.13,0.78)	0.39(0.15,0.99)
Variance of residence			0.19(0.04,0.82)
Variance of wealth index			0.02(0.01,0.15)
Variance of education			0.02(0.01,0.11)
ICC	0.338	0.092	0.11
Deviance-based chi-square	1373.60(0.001)	228.48 (0.001)	247.01 (0.0001)
${\hat{\sigma}}_e^2$	3.29		

In addition, the between-region variance ${\hat{\sigma}}_{0j}^2$ was 1.68. To examine the hypothesis that whether or not this between- regional variance would have statistically significant effect on utilization of institutional delivery, a 50:50 mixture chi-square test was employed. Because the constrained variance component test lies on the boundary of the parameter space (variance is not expected to go below zero), the likelihood ratio test statistic converges to a 50:50 mixture of chi-square distributions with 0 and 1 degree of freedom given as 0.5${\chi}_0^2$ + 0.5${\chi}_1^2$ [18, 19].

Therefore, to see whether geographical region in Ethiopia an effect in the utilization of institutional delivery care service had, the deviance-based chi-square (Table 2) is 1373.6 with average *p*-value < 0.0001. This indicates that there exists a statistically significant variation across regions for the likelihood of institutional delivery. Accordingly, the random coefficient model (model 3) was considered for predicting women’s decision about place of delivery. The random coefficient model showed that the random effects of residence and wealth index to deliver in health facility vary across regions of Ethiopia. The variance of intercept in the random slope model is 0.38, which is still large and statistically significant. Thus, there remains some regional level variance unaccounted for in the model. The between-regions variance in the effect of place of residence on institutional delivery estimated as 0.24. This suggests that the effect of place of residence may be justified in constructing the effect to be random.

### Effect sizes of factors for institutional delivery

Details of the effect sizes of factors on the odds of institutional delivery are shown in Table 2. The frequency of ANC visits was significantly associated with place of delivery. After adjusting other variables in the model, the odds of giving birth at health facility was 3 times (AOR = 3.12, 95% CI: 2.73–3.56) higher among women who had recommended four or more visits compared with those who had less than four ANC visits. The odds of delivering at health facility was higher for women residing in urban (AOR = 7.18, 95% CI: 5.10–10.12) compared rural areas. The odds of delivering at health facility among women who attend primary school was 1.77 times (AOR = 1.77, 95% CI: 1.49–2.10), secondary school was 3.79 times (AOR = 3.79, 95% CI: 2.72–5.30) and higher level was 5.86 times (AOR = 5.86, 95% CI: 3.25–10.58) higher compared to women who had no formal education, respectively.

Increasing in parity was negatively associated with the odds of delivering at a health facility. Compared to prim parous women, the odds of institutional delivery were 48 and 40% lower for women who had five or more and two to four previous births, respectively. Compared to women residing in poor households, the odds of institutional delivery were higher for women residing in with rich (AOR = 2.39, 95% CI: 1.86–3.06), and middle (AOR = 1.66, 95% CI: 1.36–2.03) wealth index households. Distance from the health facility was positively associated to the odds delivering at health facility. The women who live near to health facility were 1.13 (AOR = 1.13, 95% CI: 1.06, 1.22) times higher chance of deliver at heath facility than those living far from health facility.

## Discussion

This study attempts to identify the determinant factors of institutional delivery service utilization in Ethiopia. The result of this study showed that the level of institutional delivery was 38.9%. The result from multivariable analysis showed that ANC visits, residence, education, parity, multiple birth type, size of child at birth, and household wealth index were significantly associated factors with institutional delivery. A significant variation of institutional delivery was observed across regions. The intra-class correlation coefficient (ICC) indicates that approximately 34% of the total variation in the institutional delivery explained by the region of mothers.

Antenatal care is a proximate predictor of women’s decision on place of delivery. Women who had a minimum number of visits recommended by WHO were more likely to give birth at health facility. This is due to the fact that women who seek care for their pregnancy are more likely to seek care for their delivery. This finding is comparable with other previous studies conducted in Ethiopia and Tanzania [5, 18–21]. Antenatal care is the most favorable contact point for mothers to get more information about risks and problems that they may encounter during delivery. Women who frequently visits health facility for ANC service had already demonstrated some acceptance of the health care system and recommended by health professionals to deliver at the health institutes.

In the current study, women’s residence was significantly associated with the utilization of institutional delivery services. Women residing in urban areas had a higher likelihood of delivering at a health facility than rural residents. This finding is consistent with other previous studies conducted in sub-Sahara African countries [22–26]. Women who residing in urban areas, are more exposed to media messages, more educated and informed, and being close to health institutions. Distance to health facility and transportation problems are make less likely to give birth in health institute for mothers those residing in rural areas. Additionally, deeply rooted negative beliefs and myth regarding to institutional delivery, inadequacy health services and low infrastructure in rural Ethiopia determines women’s health seeking behavior.

Having formal education was positively associated with institutional delivery. This finding is supported with other studies conducted in Ethiopia, Ghana, Nigeria and Nepal [2, 7, 27–31]. Possible mechanisms include access to information, financial freedom and autonomy, which brings a higher level of awareness and better knowledge of delivery care services. These could be collectively influence mothers’ awareness to seek better medical services, including delivering in health facilities.

This study found that higher parity is negatively associated with institutional delivery. This result is supported by other studies [2, 11, 21, 24, 25, 32]. The odds of institutional delivery for mothers having multiple gestations were higher than those having single gestation. The odds of using institutional delivery services was about 20 and 30% lower when women had an average and small size of the child as compared to those who had a large size of the child at a time of delivery. Possible mechanisms or explanations for this result is as the size of gestation increases, the pregnancy complication may increase which enforce women to attend health facility to get safe delivery.

The wealth index is a composite measure of the cumulative living standard of a household used as a proxy for socioeconomic status. The current study revealed that women socioeconomic status is positively association with institutional delivery. This is supported by other studies [2, 30]. Household wealth has a potential to influence women’s decision regarding place of delivery, access to health care services, transportation and additional costs. Women who can afford to pay for such costs are more likely to visit health facilities.

The study found that distance from the health facility was a big problem to facility delivery. It has been shown that women who living near to health facilities is more likely to deliver at health facility than those living far from health facilities. This finding is consistent with the studies conducted in Ethiopia, Eritrea, Kenya and Nigeria [33–36]. This is due to the fact that the longer the distance to reach health facility lacks easy transportation services and unaffordable costs provide home delivery care.

The multilevel logistic regression analysis confirmed the significance of regional difference in utilization of institutional delivery in Ethiopia. The null model suggests that women with the same characteristics in different regions have different rates of institutional delivery suggesting the need for interventions to focus more at regional level followed by individual level.

There is a dearth of work on EDHS to choose a model which considers the regional variation and this work have tried. Ethiopian government have tried tremendous work to improve the health of its citizens and to reduce maternal mortality. But there is no considerable improvement compared with the countries five year back report about institutional delivery. Hence, the government have work on again on, women education, health facility coverage, ANC service, and poverty reduction.

### Strength and limitation

This study was population-based study, which involved large sample data. Multiple confounding variables were assessed using advance methodology. The cross-sectional nature of the study precludes drawing conclusions about the temporal nature of the association between the independent variables and the outcome of interest. Other limitations include recall bias and unmeasured confounders such as distance to health facility that was not captured in the 2016 EDHS.

## Conclusion

The level of institutional delivery services in Ethiopia remains low. Despite of the government report as women have improved access on health facility delivery, a considerable number of women are still giving birth at their home. Frequency of ANC visits, place of residence, women’s educational level, and household wealth index were significantly associated factors with institutional delivery. Programs aiming to improve institutional delivery in Ethiopia have to consider these factors.

## Data Availability

The datasets and materials used in this study are available upon request to the corresponding author.

## Abbreviations

ANC: Antenatal care
AOR: Adjusted Odds ratio
CSA: Central statistical agency
CI: Confidence interval
EDHS: Ethiopian demographic and health survey
ICC: Intra-class correlation coefficient
SNNPR: Southern Nations, Nationalities, and Peoples’ Region
